# *QuickStats*: Age-Adjusted Percentage* of Adults Aged ≥18 Years Who Take Prescription Medication for Depression,^†^ by Sex and Race and Hispanic Origin — National Health Interview Survey,^§^ United States, 2021

**DOI:** 10.15585/mmwr.mm7221a5

**Published:** 2023-05-26

**Figure:**
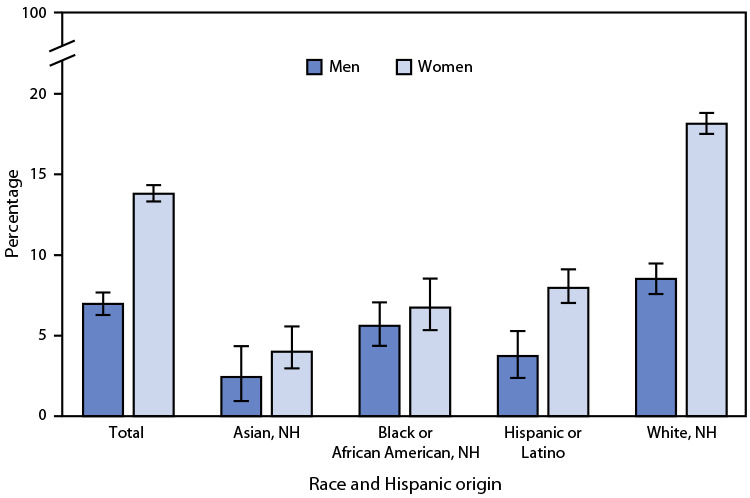
In 2021, among adults aged ≥18 years, women were more likely to take prescription medication for depression than were men (13.8% versus 7.0%). This pattern was found among non-Hispanic White (White) (18.1% versus 8.5%) and Hispanic or Latino (8.0% versus 3.7%) adults, but differences by sex were not statistically significant among non-Hispanic Black or African American (Black) (6.7% versus 5.6%) and non-Hispanic Asian (Asian) (4.0% versus 2.4%) adults. Among men, Asian adults were less likely than White and Black adults to take prescription medication for depression, but the difference compared with Hispanic adults was not statistically significant. Among women, Asian adults were the least likely to take prescription medication for depression.

**Source:** National Center for Health Statistics, National Health Interview Survey, 2021. https://www.cdc.gov/nchs/nhis/index.htm

